# Hemidesmosomal Reactivity and Treatment Recommendations in Immune Checkpoint Inhibitor-Induced Bullous Pemphigoid—A Retrospective, Monocentric Study

**DOI:** 10.3389/fimmu.2022.953546

**Published:** 2022-07-22

**Authors:** Franziska Schauer, David Rafei-Shamsabadi, Shoko Mai, Yosuke Mai, Kentaro Izumi, Frank Meiss, Dimitra Kiritsi

**Affiliations:** ^1^ Department of Dermatology, Medical Center - University of Freiburg, Faculty of Medicine, Freiburg, Germany; ^2^ Department of Dermatology, Faculty of Medicine and Graduate School of Medicine, Hokkaido University, Sapporo, Japan

**Keywords:** collagen XVII, immunosuppression, autoimmune bullous disorders, skin fragility, melanoma

## Abstract

Immune checkpoint inhibitors (ICI) induce T-cell-mediated antitumour responses. While ICI were initially successfully applied in metastasized melanoma, they are now approved for several tumour entities. Numerous autoimmune disorders have been reported to occur as adverse events of the treatment, among them bullous pemphigoid (BP), with less than 1% of the patients experiencing ICI-induced BP. This number is higher than the estimated prevalence of autoimmune bullous diseases in the general population of Germany, which lies around 0.05%. We here describe our cohort of eight patients, who developed a bullous pemphigoid under or shortly after ICI treatment. Half of them had a severe subtype (as shown by BPDAI >57) and showed a median onset of ICI-BP after 10 months of ICI initiation. Six patients had a palmar and/or plantar involvement, while oral involvement occurred in one case. All patients had linear epidermal IgG depositions in split skin in the indirect immunofluorescence. In four out of five biopsies available for direct immunofluorescence, linear IgG and C3 depositions were detected at the basement membrane, while one patient showed linear IgM staining. Moderate to high levels of FLBP180 autoantibodies were found in seven of eight cases. The disease can still be active after ICI discontinuation, while rituximab might be required for remission. Finally, four tumour samples were stained histochemically for collagen XVII (BP180), but no enhanced expression was found.

## Introduction

The use of immune checkpoint inhibitors (ICI) has profoundly shaped the treatment landscape for advanced, unresectable or metastatic melanoma in the past decade (1). The anti-CTLA-4 antibody ipilimumab became the first approved ICI agent in 2011 (2). Three years later, further therapeutics were approved, targeting the immune cell surface protein programmed cell death 1-protein (PD-1), to induce cytotoxic T-lymphocyte antitumor response (3–5). Beside melanoma treatment, ICI are in use for numerous tumour diseases, now including further skin cancers, like Merkel cell carcinoma (MCC) and squamous cell carcinoma of the skin (cSCC) (6–8). Data have shown that mortality rates decreased significantly in treated melanoma patients, proving the enormous success of ICI as cancer therapeutics (9). However, in up to 70% of ICI-treated patients a variety of immune-related adverse events (irAEs) of any grade occur, among them colitis, exanthema, hepatitis, endocrine dysfunction and others, which are the result of immune-mediated, toxic inflammatory changes in healthy tissues (10). In some cases, irAEs mimic features of classic autoimmune disorders (11). ICI-induced bullous pemphigoid (ICI-BP), which seems to be immunopathologically indistinguishable from sporadic BP, was found in around 0.6% of 5,636 patients treated for non-melanoma skin cancer (NMSC) or melanoma (12) and in up to 3.8% of 358 patients, who had cutaneous irAE during ICI treatment for melanoma (in total 2,459 patients) (13). Risk factors for BP were age above 70 years and early anti-tumour response (12). Depending on the disease severity of ICI-BP, sometimes there is the necessity for discontinuation of cancer treatment and additional systemic immunosuppression (14). A possible mechanistic explanation for ICI-BP is autoreactive T cells targeting BP180 on tumour cells, but also at the dermo-epidermal junction zone (DEJZ) of the skin. The interaction between PD-1/PD-L1-expressing B cells and PD-1+ follicular helper T cells facilitating humoral responses *via* B cell germinal centres are also being discussed as underlying mechanisms in ICI-BP (15–17). Contributing factors of altered expressions of BP180 in melanoma (18), aberrant immune response and genetic predisposition (HLA-DQB1*0301 for both melanoma and BP) appear to promote the loss of self-tolerance (19). In this study, we analysed a cohort of eight patients and characterized their BP-associated hemidesmosomal reactivity, along with the disease manifestations and treatment options for ICI-BP.

## Methods

### Patients

Between 2016 and 2022, we identified and characterized eight patients from the Department of Dermatology, Medical Center-University Freiburg, Germany. All patients were of European origin. In the study, we included patients who developed clinical manifestations of BP during or shortly after ICI treatment, which could be either first- or second-line treatment. The inclusion criteria were as follows: IgG deposition on the blister roof in indirect immunofluorescence on split skin (ssIIF); detection of autoantibodies against full-length BP180 (FLBP180) and/or BP180 NC16A or BP230; and if available a histopathological pattern matching BP and deposition of IgG and/or C3c at the DEJZ in direct immunofluorescence (DIF), according to the German S2k guideline (20). Patients with questionable immunofluorescence or histopathology results or unspecified exanthema were not included in this study. Patient records were analysed for clinical data (sex, age, disease severity based on the Bullous Pemphigoid Disease severity (BPDAI) (21), anticancer treatment characteristics and serological details of BP.

### Immunofluorescence

BP was diagnosed by DIF and serological tests, according to national and European guidelines (20, 22). FITC-labelled antibodies used for direct immunofluorescence were anti-human IgG (F0202), IgA (F0204), IgM (F0203) and C3c (F0201) from Agilent Technologies (Santa Clara, CA, USA), at a dilution of 1:200, 1:50, 1:50 and 1:500 respectively. The serological tests included ssIIF diagnostics on 8-µm cryosections of 1 mol/l NaCl split skin of normal human skin. For ssIIF, patient sera were diluted 1:10; the secondary antibody used was FITC-labelled anti-human IgG (F0202, Agilent Technologies, USA) at a dilution of 1:100. For semiquantitative analysis for circulating autoantibodies to the hemidesmosomes, BP230 and BP180 NC16A commercially available ELISA systems (MBL, Nagoya, Japan) were employed. Antibody titres above 9 U/ml are considered positive. ELISA using FLBP180 recombinant proteins was performed as previously described (23), with autoantibody titres above 4.64 U/ml being considered positive. Pictures of stainings were taken with Nikon Eclipse 80i. For visualization, the imaging software NIS-Elements was used.

### Immunohistochemistry

Formalin-fixed paraffin-embedded (FFPE) tumour samples were available from four patients (cutaneous SCC, nodal melanoma metastasis, subcutaneous melanoma metastasis, Merkel cell carcinoma), before ICI initiation. Serial sections were cut and stained with haematoxylin and eosin for routine diagnostics. After deparaffinization and head-induced antigen retrieval with citrate pH6 retrieval buffer, immunohistochemical (IHC) stainings were performed. Sections were incubated at room temperature for 1 h with an anti-collagen XVII antibody (Abcam, Cambridge, UK). Sections were analysed using the Dako REAL™ detection system, alkaline phosphatase/RED, Rabbit/Mouse (Dako, Glostrup, Denmark). Pictures of stainings were taken with a microscope Zeiss Axioscope. For visualization, the program AxioVision (Zeiss, Jena, Germany, SE64 release 4.9) was used.

## Results

### Patient Characteristics and Cancer Diagnosis

All eight patients had a definite diagnosis of BP. Seven patients were men, and their age at onset of BP ranged from 26 to 73 years. Five patients were treated for metastasized melanoma. The other patients presented with Merkel cell carcinoma (n = 1), hepatocellular carcinoma (n = 1) and squamous cell carcinoma of the skin (n = 1). Risk for highly aggressive squamous cell carcinoma in patient #7 was increased, due to the underlying recessive dystrophic epidermolysis bullosa. Six patients had ICI therapy with a PD-1 inhibitor as first-line therapy, while two patients (melanoma) received combined ICI therapy with PD-1 plus CTLA-4 inhibitors (patient #1, 3); both of them showed a complete remission (CR) of the melanoma. Patients #1–3 and #6 suffered from additional irAE during ICI therapy, like autoimmune nephritis (n = 2), hepatitis, hypophysitis and others (for exact patient characteristics, refer to **Table 1**). All these irAEs had been treated successfully with glucocorticosteroids before occurrence of BP in our patient cohort, so that no immunosuppressive/immunoregulatory treatment was required at time of BP onset. In three patients, ICI treatment was already terminated either due to CR of cancer (#2, 3) or due to progressive disease (#4). ICI was discontinued due to the onset and severity of BP in cases #5–8, and two of them experienced progressive disease (#6, 7).

**Table 1:** Patient characteristics and clinical features.

No.	Sex/age at cancer onset	Cancer	Oncologic treatment	Treatment response	Interval of BP onset after last ICI initiation	Age at BP onset	BP phenotype	BPDAI	BP treatment	BP duration	Other irAE
1	M/67	MM	P 2nd line (17 cycles)	CR	12 months	73	Pruritic, non-bullous, palmoplantar	>57 (sev)	tGCS, sGCS dapsone rituximab	12 months	Nephritis Hypophysitis
2	M/50	DM	P 1st line (24 cycles)	CR	15 months	72	Urticarial, bullous, palmar	49 (mod)	tGCS, sGCS dapsone rituximab	9 months	Vitiligo Thyreoiditis
3	M/51	NM	N 2nd line (32 cycles)	CR	21 months	62	Bullous postinflammatory milia, plantar	47 (mod)	tGCS, sGCS	2 months	Nephritis Hepatitis Alopecia Areata totalis
4	M/77	NM	P 1st line (24 cycles)	PD	15 months	80	Urticarial, bullous, palmoplantar	>57 (sev)	tGCS, sGCS docycycline dapsone	19 months	None
5	M/69	MUP	N 1st line (5 cycles)	PR	9 months	70	Localized, bullous	NA (mild)	tGCS, sGCS dapsone	6 months	None
6	F/64	MCC	N 1st line (2cycles)	CR	2 months	65	Localized, plantar	6 (mild)	tGCS	2 months	Oral lichen planus
7	M/26	SCC	C 2nd line	PD	3 months	26	Bullous	>57 (sev)	tGCS	6 months	None
8	M/60	HCC	N-L 1st line (5 cycles)	CR	6 months	60	Urticarial, bullous, palmoplantar, oral mucosa	>57 (sev)	tGCS, sGCS doxycycline	Ongoing	None

MUP, melanoma of unknown primary; C, cemiplimab; DM, desmoplastic melanoma; F, female; HCC, hepatocellular carcinoma; M, male; MCC, Merkel cell carcinoma; MM, malignant melanoma; N, nivolumab; NM, nodular malignant melanoma; N-L, nivolumab–lenvatinib; P, pembrolizumab; SCC, squamous cell carcinoma; tGCS, topical glucocorticosteroids; sGCS, systemic glucocorticosteroids.

### BP180 Immunohistochemistry in Tumour Samples

**Figure 1:**
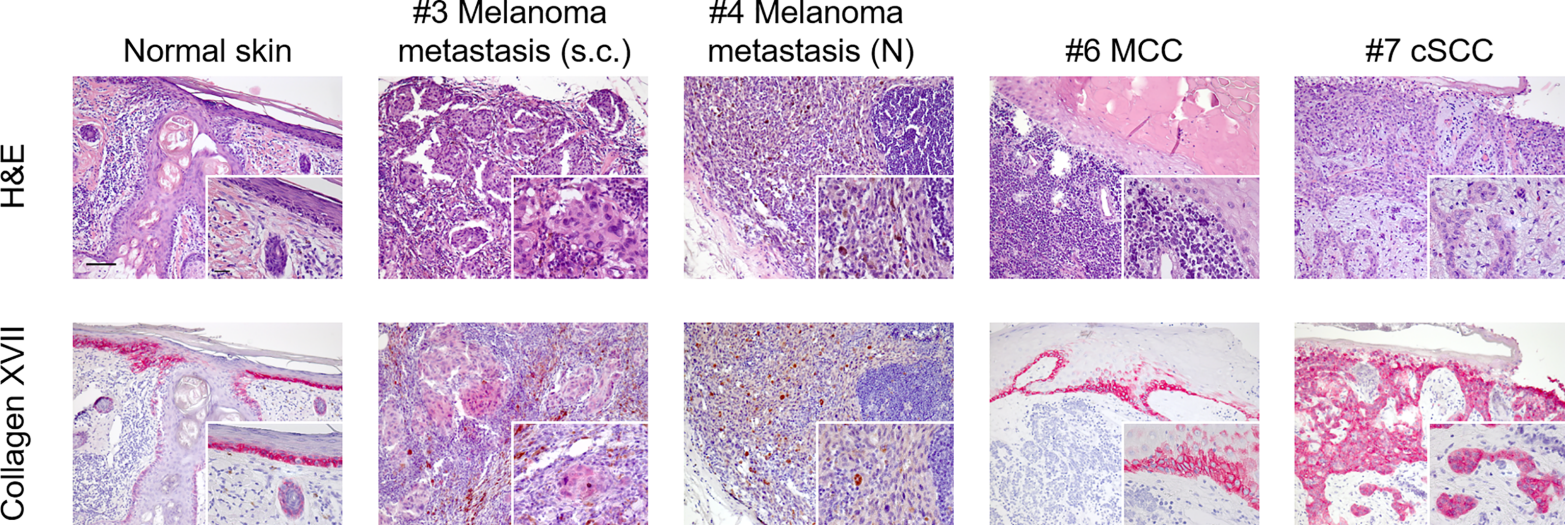
Immunohistochemistry staining for collagen XVII (BP180) in malignant tumours of the skin. Using a rabbit monoclonal antibody (Abcam, clone: EPR14758) to collagen XVII (BP180), we stained formalin-fixed paraffin-embedded tissue sections of normal skin and tumour tissue available from a subgroup of our patient cohort. Upper line shows H&E stainings. Lower line shows staining for collagen XVII (BP180). Staining for collagen XVII in normal skin shows a physiological intercellular distribution in the basal layers of epidermal keratinocytes and following the adnexal structures into the deeper dermis. Nearly all tumour cells of the cutaneous squamous cells carcinoma (cSCC) showed strong positive staining for collagen XVII (patient #7). No positive staining was seen in melanoma cells of the nodal metastasis of patient #4 or in the Merkel cell carcinoma (MCC) cells of patient #6. In the latter, positive staining could only be detected in regions of physiological epidermis. Finally, weakly positive staining was found in melanoma cells of a subcutaneous metastasis of patient #3. Scale bar = 100 µm; insert scale bar = 25 µm.

Four formalin-fixed, paraffin-embedded (FFPE) tumour biopsies were available for immunohistochemistry stainings taken prior to ICI treatment: a cutaneous SCC of patient #7, a subcutaneous melanoma metastasis of patient #3, a nodal melanoma metastasis of patient #4 and the primary Merkel cell carcinoma of patient #6. To evaluate if tumour cells express high levels of collagen XVII (BP180), thus possibly triggering BP by changing the antigen abundance and localization, we stained tumour sections with a recombinant monoclonal antibody against BP180. Normal human skin served as a positive control and showed a physiological intercellular expression of BP180 in the basal keratinocyte layers of the epidermis (**Figure 1**). Nearly all tumour cells of the cSCC of patient #7 showed a strong expression of BP180, also in keratinocytes above the basal layers. In the subcutaneous melanoma metastasis of patient #3, we found only a few tumour nests showing a weak expression of BP180. Finally, tumour cells of the nodal melanoma metastasis of patient #4 and of the Merkel cell carcinoma of patient #6 were clearly negative for BP180. In the latter, positive BP180 staining was only detectable in parts of healthy adjacent epidermis (**Figure 1**). Thus, it seems that tumours of non-epidermal/keratinocyte origin do not overexpress BP180 per se.

### ICI-BP—Clinical Presentation

**Figure 2:**
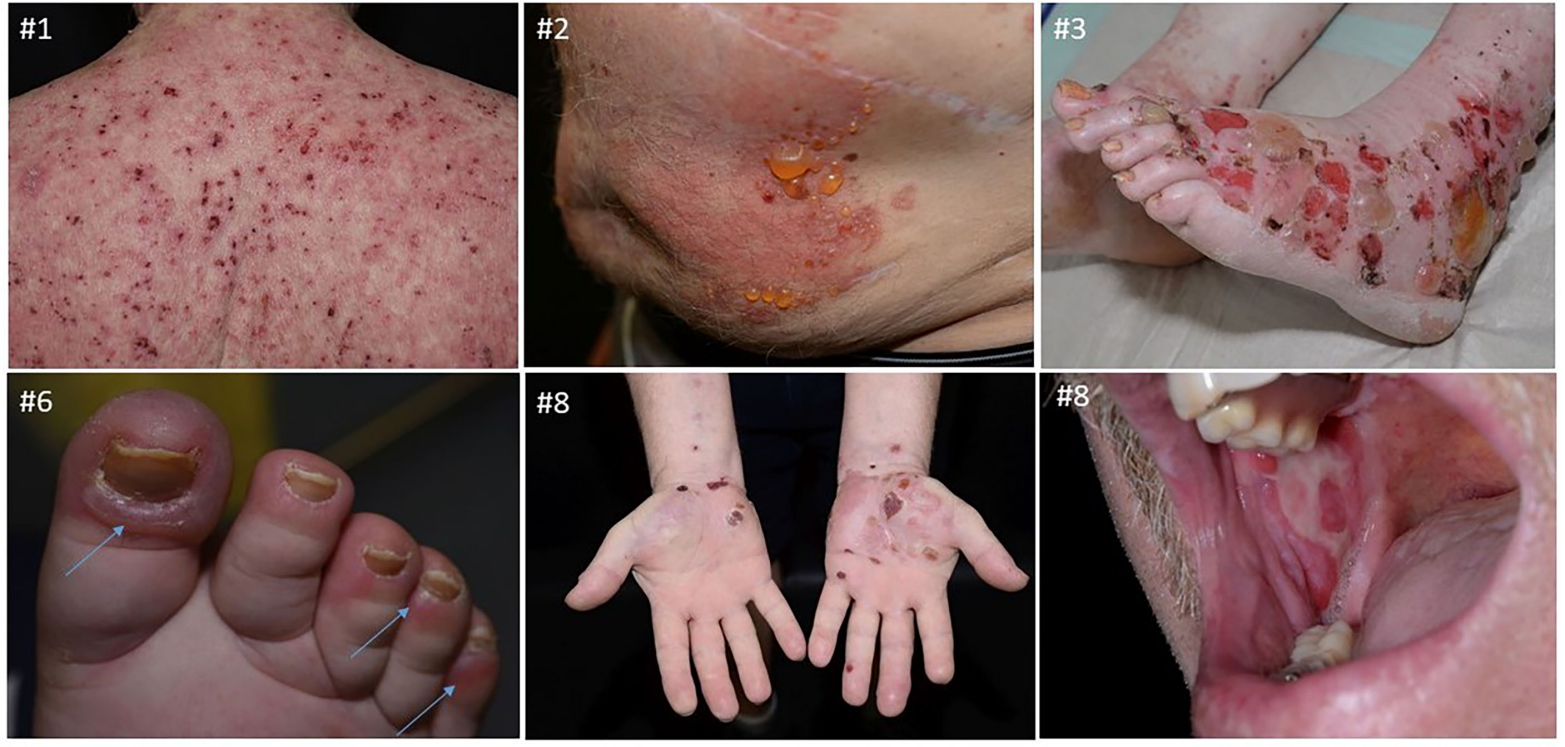
Clinical presentation of selected patients of the cohort, #1 shows excoriated, haemorrhagic erosions on erythematous ground on the back, #2 shows urticarial plaques with tense blisters on the abdomen, #3 presents with tense and eroded blisters on erythematous ground on the dorsum of the feet and lower leg, #6 shows periungual tense, dyshidrosiform blisters of the digitus I, IV and V and #8 shows tense, dyshidrosiform blisters on the palms of the hands and erosions on the right buccal mucosa.

All patients had a severe, sudden onset of intense pruritus. An evaluation of disease severity with the BPDAI score revealed the following patient groups: a group with mild (two patients), with moderate (two patients) and with severe (four patients) skin involvement. Time of BP onset ranged from 2 to 21 months (median 10 months) after ICI treatment start. Patients #1 and #4 had a non-bullous, pruritic phenotype initially, gradually evolving into urticarial plaques (#1) and tense blisters (#4) in the course of time. The other patients presented with a bullous phenotype at disease onset. Patient #8 was the only one showing involvement of the oral mucosa, additionally to generalized skin blistering (**Figure 2**). Furthermore, six out of eight patients (75%) had the picture of dyshidrosiform BP with tense blisters of the palms and/or soles. Patient #2 showed milia after healing.

### ICI-BP—Immunofluorescence and Serological Results

**Figure 3:**
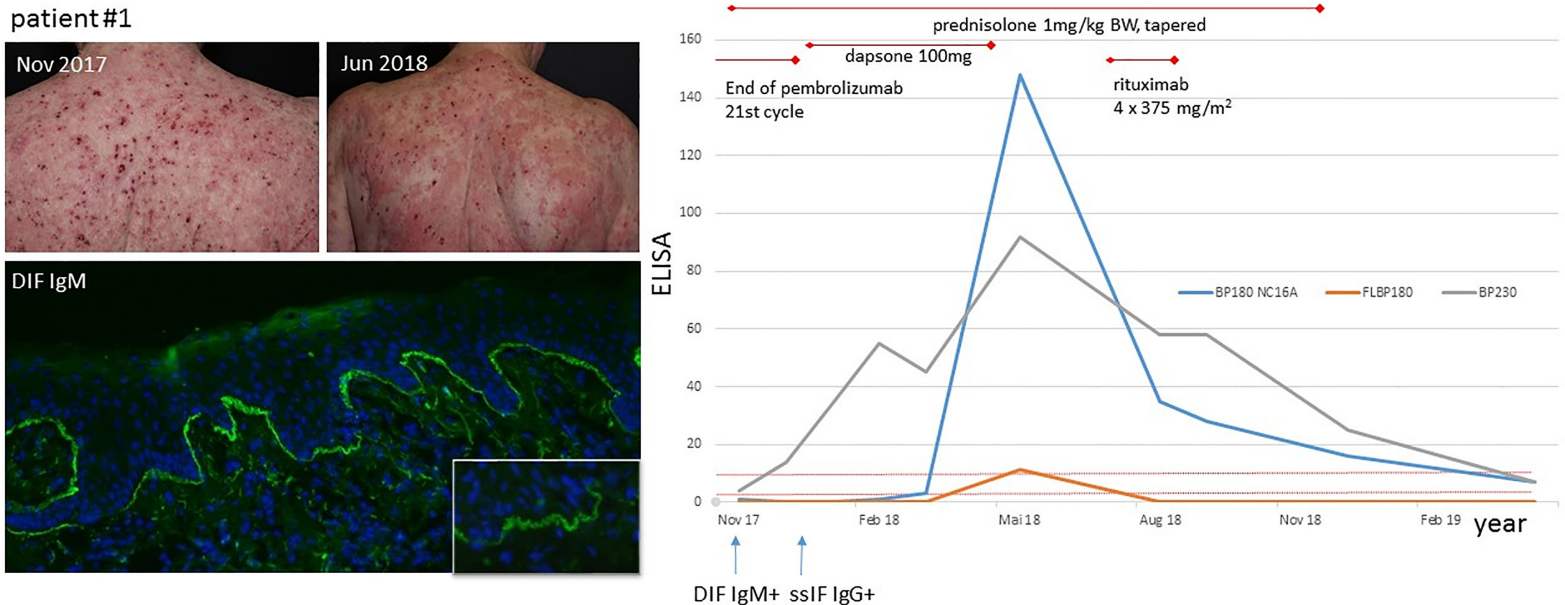
Clinical presentation and BP progression of patient #1. Upper left picture (Nov 2017): eroded, haemorrhagic crusts on erythematous ground, which changed to urticarial plaques in June 2018 (upper right picture), when BP180 NC16A ELISA became positive. Direct immunofluorescence (lower left picture) with linear IgM n-serrated deposition at DEJZ of patient #1. Serological course of patient #1: initial positivity of BP230, followed by FLBP180 and BP180 NC16A in the course of time. Serological remission after therapy with rituximab in February 2019.

All patients had a positive IgG deposition at the blister roof in ssIIF. DIF was performed in five out of eight patients. Four patients had linear IgG and C3 depositions along the DEJZ in DIF. Patient #1 had at the initial presentation IgM depositions at DEJZ exclusively, while serological analysis was negative. Serological analysis performed at a later time point revealed IgG reactivity to BP230, FLBP180 and BP180 NC16A, as shown in **Figure 3**. Moderate to high FLBP180 autoantibody titres were found in seven cases. Notably, in patient #3 and patient #6 no other autoantibodies were identified. Patients #1–3 had elevated IgE levels, which were above 2,100 kU/l in patient #1. Blood eosinophilia was present in five cases and increased compared to baseline or during ICI treatment at the time of BP diagnosis and was therefore interpreted as BP-associated (**Table 2**). In patient #3, an increased absolute eosinophil blood count (AEC) was already evident during the last cycles of nivolumab therapy and was timely not related to BP diagnosis.

**Table 2:** Immunofluorescence characteristics of the ICI-BP cohort—some of these samples were taken at different time points or at disease maximum.

No	DIF	ssIIF(roof side)	FLBP180	BP180 NC16A	BP230	IgE (kU/l)	Blood eosinophilia (thousands/µl)
1	IgM +	IgG +	11.21	148	92	1,063	0.55
2	IgG +, C3 +	IgG +	16.19	39	10	233	2.31 (BP)
3	IgG +, C3 +	IgG +	86.23	n	n	141	0.85 (ICI)
4	IgG +, C3 +	IgG +	47.15	118	n	141	3.41 (BP)
5	NA	IgG +	n	25	n	335	0.32
6	NA	IgG +	26.88	n	n	NA	0.37
7	NA	IgG +	7.74	10	15	21	3.57 (ICI)
8	IgG +, C3 +, discrete IgM	IgG +	105.79	15	2	NA	0.45

n, negative; ssIIF, split skin of indirect immunofluorescence; FLBP180, cutoff <4.64 U/ml; BP180, NC16A cutoff <9 U/ml; BP230, cutoff < 9 U/ml; IgE positive > 100 kU/l, blood eosinophilia cutoff > 0.44 thousands/µl.

### BP Treatment

All patients were treated with topical clobetasol propionate and systemic glucocorticosteroids for several weeks (prednisolone up to 1 mg/kg body weight (BW), followed by tapering according to the EADV/EDF guideline recommendations). In four patients (#1, 2, 4, 5) with at least moderate disease (BPDAI ≥20), additional dapsone therapy with up to 100 mg per day was initiated, leading to BP remission in two of them.

For the remaining two patients with treatment resistance to glucocorticosteroids combined with dapsone, treatment with rituximab was decided. Patient #8 initially refused systemic treatment, desiring only mild immunomodulatory therapy. However, low-dose prednisolone together with doxycycline 200 mg per day showed no sufficient effect. In patients #1–7, clinical and serological remission was achieved after 2 to 19 months of disease duration (median: 8 months).

## Discussion

We identified eight patients who developed ICI-BP in 2016 to 2022 in a single centre in Germany. Our data confirm the rarity of ICI-BP with a reported incidence of 1.5% in 533 institutional ICI treatments during this period. This number is definitely higher than the prevalence of 0.05% for bullous autoimmune diseases in Germany (24).

In our cohort, the onset of BP ranged from 2 to 21 months (median 10 months) after start of ICI treatment. In another recently published German multicentre study with 12 patients, BP occurred between 3 and 74 weeks (median 23 weeks) which is half the time in our cohort (14). Since both patient cohorts are rather small, more studies are required to define the median time of BP onset.

Cutaneous irAEs of any kind usually manifest within the first month of ICI therapy, but they can develop at any time of treatment or even after stopping therapy (25). Linear, epidermal IgG depositions were present in all of our patients in the ssIIF, while DIF showed linear IgG and C3 staining at DEJZ in four patients and was not performed in three patients. Intriguingly, in patient #1 we observed an IgM deposition with a clear n-serrated pattern along the DEJZ in DIF, as an initial immunological reaction. At first, IgM deposits could not be assigned to BP diagnosis, since ssIIF and commercial IgG ELISA were negative. The subsequent clinical and serological course confirmed the diagnosis of BP with gradual epitope spreading and identification of IgG deposits. Thus, the initially identified IgM deposits were possibly part of a prodromal stage of BP. We stained both IgG1 and IgG4 in the patient’s initial skin biopsy, since both have been reported to be important for BP induction, with or without need of complement fixation, respectively, but they were negative in our case (26). The first cases of IgM pemphigoid were published recently (27); however, the pathogenicity of IgM autoantibodies remains controversial. In the aforementioned article, IgM immunoreactivity was detected through immunoblot diagnostics against fragments of the C-terminus of the ectodomain (Col17ec3) of collagen XVII in three patients. *Ex vivo* complement fixation tests showed the inability of IgM autoantibodies to activate complement, which is considered to be the main pathogenetic mechanism in BP (27). Patient #8 of our cohort showed a linear IgM deposition besides IgG and C3 at the DEJZ, which has been also described in up to 17% of BP patients investigated by Moriuchi et al. (28, 29). In a patient with exclusive IgM staining at DEJZ, detailed super-resolution imaging detected deposits close to the NC1 domain of collagen VII, suggesting once more that IgM antibodies possibly target the C-terminus of BP180 (28). Clinical manifestations included papules or urticarial lesions, without blisters or mucosal involvement. Our patient #1 did not have any blisters as well. Clinically we found eroded, haemorrhagic and crusted plaques and an increased inflammatory reddish ground on the whole skin (**Figure 3**). Interestingly, it was evident in our cohort that the majority of the patients had a palmoplantar manifestation. Patient #6 had exclusively periungual blisters. Dyshidrosiform pemphigoid is a unique type of BP resembling dyshidrotic eczema. This type of BP can be drug-related, and bullae may subsequently spread to the whole body (30). It also has been found related to ICI treatment in another case (31). It would be interesting to further assess the relevance for IgM autoantibodies, specifically in ICI-BP.

In sporadic BP, usually the autoantibodies target BP230 and in 90% of cases the juxtamembranous extracellular NC16A domain (32). In a multicentre study, autoantibodies against NC16A were observed at the initial stages of the disease, followed by epitope spreading in some cases (33). The role of epitope spreading was specifically examined in BP induced by prolonged use of dipeptidyl peptidase-4 inhibitors (DPP4i) (34). The detection of FLBP180 autoantibodies has been described in a non-inflammatory, mild BP phenotype, frequently associated with DPP4i treatment for type 2 diabetes (23, 34). Patient #1 is exemplary for the epitope spreading with an initial negativity of BP180 NC16A, which became positive during BP exacerbation and the continued therapy (**Figure 3**). Patients #3 and #6 had FLBP180 autoantibodies exclusively, notably with diverse clinical manifestations. None of the patients in our cohort took DPP4i. BP180 (collagen XVII) is a hemidesmosomal transmembrane glycoprotein, expressed in basal keratinocytes. BP180 is down-regulated in mature keratinocytes, when they migrate through the upper layers of the epidermis, but is found reexpressed in SCCs indicating dedifferentiation (18, 35). In head and neck squamous cell carcinoma (HNSCC), an increased BP180 expression was associated with a more aggressive cancer type and a poorer outcome (36). Interestingly, BP180 has been shown to be expressed in malignant, but not in benign, melanocytic tumours and it can mediate antibody-induced melanoma apoptosis (18). In two out of five melanoma patients in our cohort, tumour tissue was available for IHC staining for BP180 expression. We could only detect a weak BP180 staining in some tumour nests of a subcutaneous melanoma metastasis, whereas no melanoma cells of a nodal metastasis showed positivity. No BP180 staining was observed in the Merkel cell carcinoma cells. Although we could not confirm strong BP180 staining in melanoma cells, the sample size was too low to draw a conclusion. Further studies with larger sample sizes should clearly address this issue. Finally, a strong BP180 expression was detected in nearly all cells of our SCC patient. The high expression may be in line with the moderately differentiated phenotype and the clinically big tumour size, indicating a more aggressive cancer as shown in HNSCC (36). Whether increased BP180 expression in skin tumours might trigger induction of ICI-BP remains to be further investigated.

**Figure 4:**
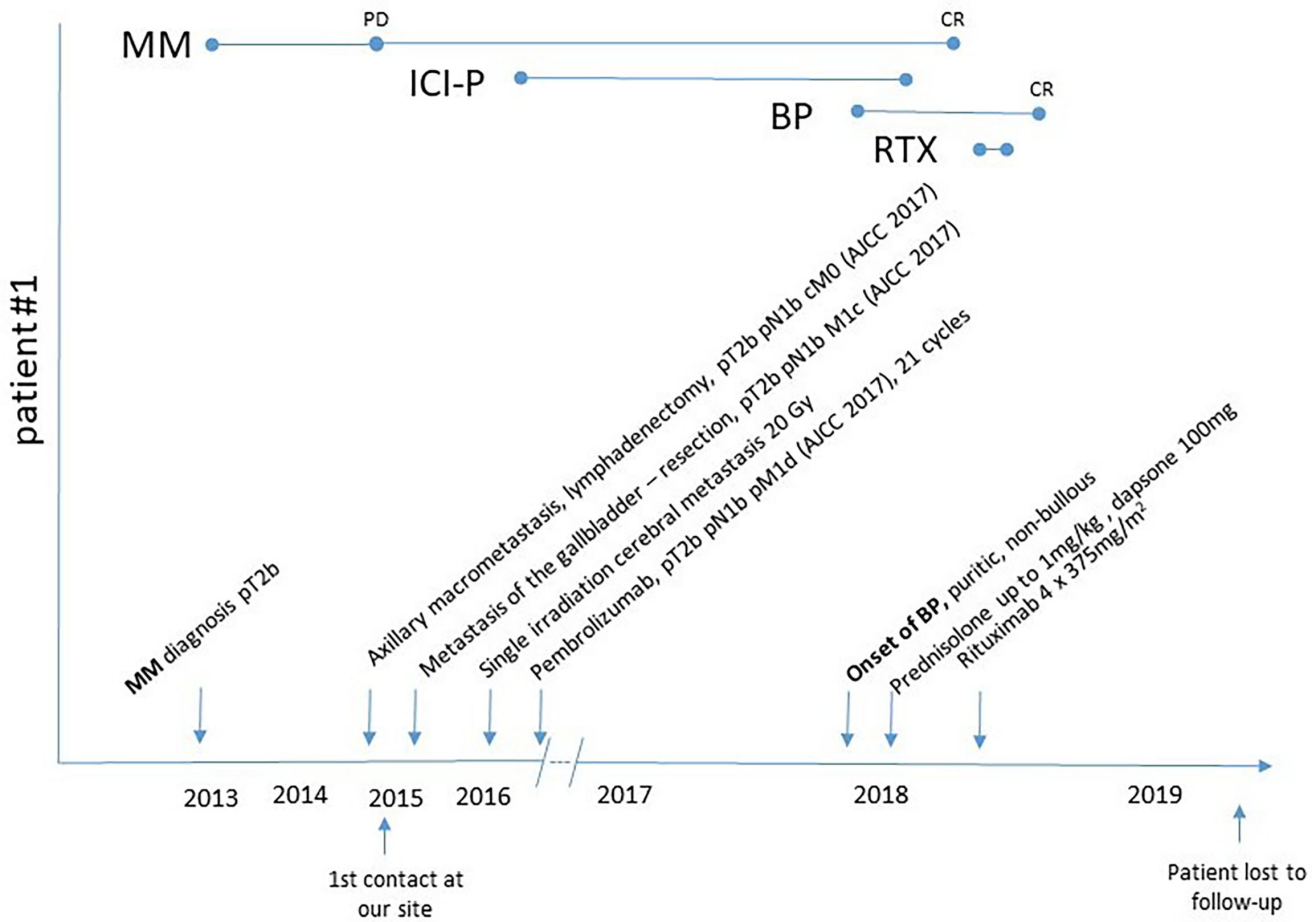
Clinical course of patient #1: Disease onset of melanoma in 2013, axillary lymph node macrometastasis in 2015, pembrolizumab initiation in 2016 because of cerebral metastasis, onset of pruritic exanthema in the end of 2017 and diagnosis of BP in 2018.

irAEs require early detection and appropriate intervention, considering their impact on patients’ morbidity rather than mortality. For frequent irAEs, organ system-based management algorithms have been developed depending on the grade of severity (11). ICI-BP should be treated according to the guidelines for spontaneous BP, which includes highly potent topical glucocorticosteroids for mild to moderate diseases, combined with a systemic treatment in severe cases (20). Treatment strategies which do not directly inhibit antitumour-specific T cells are certainly favoured to avoid inhibition of the antitumour effect of the ICI. A systemic therapy for BP is recommended to be initiated with a dose of 0.5 mg per kg per day of prednisolone equivalent, potentially in combination with adjuvant immunosuppressive/immunomodulatory therapy, like dapsone or doxycycline. Immunosuppressive therapies with agents like azathioprine or mycophenolate mofetil are controversial, because strong and long-lasting immunosuppression may trigger tumour progression leading to a reduced overall survival (37–39). We started topical and oral glucocorticosteroids and dapsone in four out of eight patients, but therapy had to be extended to rituximab in two cases (**Figure 4**). Sowerby et al. have reported a case of nivolumab-induced BP, which was completely resolved after four doses of rituximab 375 mg/m^2^ once weekly (40). Winkler et al. published a case series of seven patients with metastasized melanoma, which were treated individually with rituximab after prior insufficient response to standard-of-care treatment. Five patients achieved stable disease, and two patients showed tumour progression (41). In both our patients, BP resolved quickly after rituximab administration and serological activity ended after 12 months. Methotrexate (MTX) use sometimes seems controversial. In general, it may be of use in the treatment of sporadic BP as a steroid-sparing agent and may be considered as a first-line treatment for ocular mucous membrane pemphigoid (20). MTX is approved to be used in the treatment of acute lymphocytic leukaemia and other types of solid cancer (brain tumours, breast cancer, lung cancer, lymphomas, esophagogastric carcer, local osteosarcoma, prostate and bladder cancers) (42). Shi et al. treated five of 14 ICI-BP patients in their cohort with MTX in variable doses (7.5 to 25 mg per week) with a good response in three of them (13). Dupilumab therapy has been recently proposed for sporadic BP and is currently being investigated in a multicentre phase III study; thus, it might also be useful for ICI-BP (13, 43).

The increase of the AEC in peripheral blood has been reported to be a positive predictive immune marker in advanced melanoma patients, probably due to facilitated T-cell recruitment (44, 45). Treatment with CTLA-4 inhibitors alone or PD-1 inhibitors in combination with intralesional interleukin-2 in melanoma patients may lead to an AEC increase, associated with improved progression-free and overall survival (44, 45). Peripheral eosinophilia has been reported in 50%–60% of patients with BP and correlated positively with disease severity (46, 47). Thus, it is conceivable that ICI-BP may positively correlate with tumour response, as observed in two of our patients. Others had progressive disease (patient #7, SCC) or partial response (patient #5, MM) but with fewer treatment cycles.

ICI-BP has been described as an adverse event of the treatment, as similarly shown for other drugs DPP4i-associated BP/drug-associated BP. It is conceivable that the occurrence of BP in cancer patients may not only be triggered by ICI treatment, since higher age and neoplasia have also been shown to be associated with higher BP incidences (17, 48). Nonetheless, in a monocentric study with 260 patients with metastatic melanoma treated with BRAF inhibitors, none of them developed BP (49, 50). This is an indicator for the causal role of ICI in BP occurrence. Examination of the causality of ICI for BP induction with *in vitro* approaches is still missing. Prospective data for hemidesmosomal autoantibodies in cancer patients prior to ICI are also not available; a cohort study addressing the relevance of pretreatment autoantibodies using ELISA and IIF would be of interest. In addition, the risk for BP increases with age; in a German healthy blood donor study with 7,063 plasma samples, 0.88% of sera reacted to desmosomal and/or hemidesmosomal structural skin proteins in indirect immunofluorescence, with predominant detection of BP180 NC16A (0.52%) over BP230 (0.04%) autoantibodies with commercial ELISA tests (51). These patients had no clear clinical signs of BP, but the question remains, whether they have a higher predisposition to develop the disease eventually.

Whether specific patients are more prone to develop ICI-BP is also still elusive. In pemphigus, the recognition of self-peptides in association with distinct HLA class II alleles by antigen-activating T cells has been suggested to be central for loss of immunological tolerance. In that sense, peptide-MHC class II multimer technology approaches may be able to detect and monitor autoreactive CD4+ T cells in patients, before ICI treatment and when BP lesions occur (52). It would be worth considering to use the abovementioned *in vivo* and *in vitro* approaches to identify patients at risk and treat them as early as possible.

So far, only bullous dermatosis and not ICI-BP is listed in the CTCAE, version 5.0. Our data clearly suggest that patients who experience an abrupt onset of pruritus, combined with exanthematic (eczematous, urticarial, excoriated) or bullous eruptions, should be screened early with BP-specific diagnostics. Topical glucocorticosteroids are sufficient in mild cases, but additional immunosuppressive agents are frequently required, while rituximab represents a good treatment option for severe cases.

## Data Availability Statement

The raw data supporting the conclusions of this article will be made available by the authors, without undue reservation.

## Ethics Statement

The studies involving human participants were reviewed and approved by Ethics Committee Freiburg No. 213/15. The ethics committee waived the requirement of written informed consent for participation.

## Author Contributions

FS, DR-S and DK had full access to all of the data in the study and take responsibility for the integrity of the data and the accuracy of data analysis. FS and DR-S contributed equally to the article. All authors read, revised and approved the manuscript.

## Funding

FS reported funding from the Berta-Ottenstein-Programme for Advanced Clinician Scientists, Faculty of Medicine, University of Freiburg. DK reported funding from the Berta-Ottenstein-Programme for Advanced Clinician Scientists, Faculty of Medicine, University of Freiburg, the German Research Foundation (through SFB1160 project ID: B03, SFB1479 project ID: 441891347 and KI1795/2-1). DRS was supported by the clinician scientist program Excellent Clinician Scientists in Freiburg-Education for Leadership (EXCEL at the Medical Center University of Freiburg, Faculty of Medicine, University of Freiburg, Germany), funded by the Else Kröner-Fresenius-Stiftung (funding number: EXCEL2016_Kolleg.15). The funders were not involved in the study design, collection, analysis, interpretation of data, the writing of this article or the decision to submit it for publication.

## Conflict of Interest

FM served as a consultant and/or has received honoraria from Novartis, Roche, Bristol-Myers Squibb, Merck Sharp & Dohme, Pierre Fabre, and Sanofi Genzyme and travel support from Novartis, Sunpharma and Bristol-Myers Squibb, outside the submitted work. DK served as a consultant and/or has received honoraria from Amryt Pharma, UCB, Novartis, Fibrx Derm and Colzyx.

The remaining authors declare that the research was conducted in the absence of any commercial or financial relationships that could be constructed as a potential conflict of interest.

## Publisher’s Note

All claims expressed in this article are solely those of the authors and do not necessarily represent those of their affiliated organizations, or those of the publisher, the editors and the reviewers. Any product that may be evaluated in this article, or claim that may be made by its manufacturer, is not guaranteed or endorsed by the publisher.
